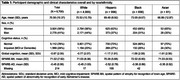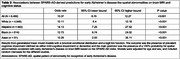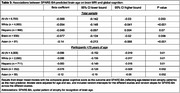# Expanding the Use of Machine Learning ADRD Indices to Diverse Neuroimaging Samples from the CHARGE Consortium

**DOI:** 10.1002/alz70856_103021

**Published:** 2025-12-26

**Authors:** Sokratis Charisis, Sachintha Ransara Brandigampala, Di Wang, Susan R. Heckbert, Jose Gutierrez, Tatjana Rundek, David M Martinez, Timothy M. Hughes, Claudia L Satizabal, William T Longstreth, Charles S. DeCarli, Derek Archer, Christos Davatzikos, Timothy J. Hohman, Sudha Seshadri, Mohamad Habes

**Affiliations:** ^1^ Glenn Biggs Institute for Alzheimer's & Neurodegenerative Diseases, University of Texas Health Sciences Center at San Antonio, San Antonio, TX, USA; ^2^ University of Texas Health Science Center at San Antonio, San Antonio, TX, USA; ^3^ UT Health San Antonio, San Antonio, TX, USA; ^4^ University of Washington, Seattle, WA, USA; ^5^ Taub Institute for Research on Alzheimer's Disease and the Aging Brain, New York, NY, USA; ^6^ Columbia University Irving Medical Center, New York, NY, USA; ^7^ University of Miami Miller School of Medicine, Miami, FL, USA; ^8^ Evelyn F. McKnight Brain Institute, Miami, FL, USA; ^9^ Wake Forest University School of Medicine, Winston‐Salem, NC, USA; ^10^ The Framingham Heart Study, Framingham, MA, USA; ^11^ University of Texas Health San Antonio, San Antonio, TX, USA; ^12^ Department of Neurology & Imaging of Dementia and Aging Laboratory, University of California, Davis, Davis, CA, USA; ^13^ Vanderbilt Memory and Alzheimer's Center, Institute for Medicine and Public Health, Vanderbilt University Medical Center, Nashville, TN, USA; ^14^ University of Pennsylvania, Philadelphia, PA, USA; ^15^ Vanderbilt Memory & Alzheimer's Center, Vanderbilt University Medical Center, Nashville, TN, USA; ^16^ Vanderbilt Genetics Institute, Vanderbilt University Medical Center, Nashville, TN, USA; ^17^ Glenn Biggs Institute for Alzheimer's & Neurodegenerative Diseases, University of Texas Health Sciences Center at San Antonio, San Antonio, TX, USA; ^18^ Neuroimage Analytics Laboratory and Glenn Biggs Institute Neuroimaging Core, Glenn Biggs Institute for Neurodegenerative Diseases, University of Texas Health San Antonio, San Antonio, TX, USA

## Abstract

**Background:**

Previous studies have leveraged machine learning (ML) analytics to detect brain atrophy patterns that are specific to Alzheimer's disease (AD) and to distinguish them from structural changes related to brain aging. These tools have been primarily developed and validated in White individuals; therefore, their applicability in diverse samples remains uncertain. Herein, we explore the generalizability of ML indices in a racially and ethnically diverse neuroimaging sample.

**Method:**

MRI data from four studies (FHS, NACC, CHS, NOMAS) including a total of 6,700 scans were processed using robust statistical harmonization pipelines to remove scanner‐related differences and were utilized to derive ML indices that quantify AD‐like (SPARE‐AD) and aging‐related (SPARE‐BA) brain atrophy patterns. A global cognitive score was computed as a weighted sum of each study's cognitive tests, using the first principal component loadings from a PCA containing the normalized scores of the cognitively normal participants as weights. We examined the associations of SPARE‐AD with cognitive impairment (defined as prevalent mild cognitive impairment or dementia) and the associations of SPARE‐BA with global cognition in the total sample and across race and ethnicity using mixed effects models adjusted for age and sex.

**Result:**

Mean age (SD) was 70.58 (10.97) years; 59% of participants were women (Table 1). A >50% probability for spatial abnormalities consistent with early AD on brain MRI based on the SPARE‐AD index was associated with a tenfold increase in the odds for cognitive impairment (OR[95%CI] = 10.37[8.76,12.27]). Similar associations were observed across race and ethnicity (Table 2). A 1‐year increase in SPARE‐BA‐predicted brain age was associated with an 8.6% of a standard deviation decrease in global cognitive score (β[95%CI] = ‐0.086[‐0.142,‐0.03]). This association was not significant in Hispanic individuals (Table 3). In individuals <75 years of age, associations between SPARE‐BA and global cognition were more consistent across race and ethnicity (Table 3).

**Conclusion:**

Robust harmonization techniques and ML model training using diverse neuroimaging datasets can facilitate the expansion of ML indices to different racial and ethnic groups. Older Hispanic individuals may have a different relationship between aging‐related brain atrophy patterns and cognition, which might indicate differences in underlying neuropathologies or other unidentified factors.